# Dr. Frederick J. Harter

**Published:** 1917-03

**Authors:** 


**Dr. Frederick J. Harter,** P. & S. (Columbia) 1880, died at his home in Herkimer, N. Y., of pneumonia Jan. 20, aged 61.

